# Novel compound heterozygous *ASXL3* mutation causing Bainbridge-ropers like syndrome and primary IGF1 deficiency

**DOI:** 10.1186/s13633-017-0047-9

**Published:** 2017-08-04

**Authors:** Dinesh Giri, Daniel Rigden, Mohammed Didi, Matthew Peak, Paul McNamara, Senthil Senniappan

**Affiliations:** 1 Institute in the Park, Alder Hey Children’s NHS Foundation Trust, University of Liverpool, Eaton Road, Liverpool, UK; 2 Institute of Intergrative Biology, University of Liverpool, Liverpool, UK; 3 Department of Paediatric Endocrinology, Alder Hey Children’s NHS Foundation Trust, Liverpool, UK; 4 NIHR Alder Hey Clinical Research Facility for Experimental Medicine, Liverpool, UK

**Keywords:** *ASXL3*, Bainbridge-Ropers syndrome, IGF-1 deficiency

## Abstract

**Background:**

De novo truncating and splicing mutations in the additional sex combs-like 3 (*ASXL3*) gene have been implicated in the development of Bainbridge-Ropers syndrome (BRPS) characterised by severe developmental delay, feeding problems, short stature and characteristic facial features.

**Case presentation:**

We describe, for the first time, a patient with severe short stature, learning difficulties, feeding difficulties and dysmorphic features with a novel compound heterozygous mutation in *ASXL3*.Additionally the patient also has primary insulin like growth factor-1 (IGF1) deficiency. The mutations occur in exon 11 and proximal part of exon 12 and are strongly conserved at the protein level across various species. *In-silico* analyses using PolyPhen-2 and SIFT predict the amino acid substitutions to be potentially deleterious to the protein function. Detailed bioinformatics analysis show that the molecular defects caused by the two compound heterozygous mutations synergistically impact on two points of the molecular interaction network of *ASXL3.*

**Conclusion:**

We hypothesise that *ASXL3* potentially has a role in transcriptional activation of *IGF1* involved in signalling pathways that regulate cell proliferation and growth, which could be contributing to short stature encountered in these patients.

## Background

The use of next generation sequencing in children with undiagnosed or unidentified syndromic disorders is becoming more popular in recent years, increasing the diagnostic ability and discovery of novel genes and mutations contributing to novel clinical phenotypes.

Bainbridge-Ropers syndrome (BRPS: OMIM #615485) was described for the first time by Bainbridge and his colleagues in the year 2013 [1]. BRPS is caused by de-novo truncating mutations in the additional sex combs-like 3 (*ASXL3)* gene giving rise to characteristic phenotypic features such as short stature, severe intellectual deficit, feeding difficulties, failure to thrive and cranio-facial features. BRPS has been reported in 27 patients in the literature so far [1–4]. The majority of the patients had frameshift or truncating mutations in *ASXL3*. One patient has been reported to have a splicing mutation in *ASXL3* resulting in BRPS [3].

Bohring-Opitz syndrome (BOS: OMIM#605039) is a developmental syndrome characterised by a severe intellectual deficit, distinct posture and cranio-facial abnormalities, feeding problems and failure to thrive [5]. BOS is caused by de novo truncating mutations in *ASXL1*, which belongs to the same family as *ASXL3* [5]. BOS and BRPS have been found to have some overlap of their clinical phenotypes.

We describe, for the first time, a patient, with severe short stature secondary to IGF1 (Insulin Growth Factor 1) deficiency, developmental delay, intellectual deficit, cranio-facial abnormalities due to a novel compound heterozygous mutation in *ASXL3* identified by whole exome sequencing.

## Case presentation

**Fig. 1:**
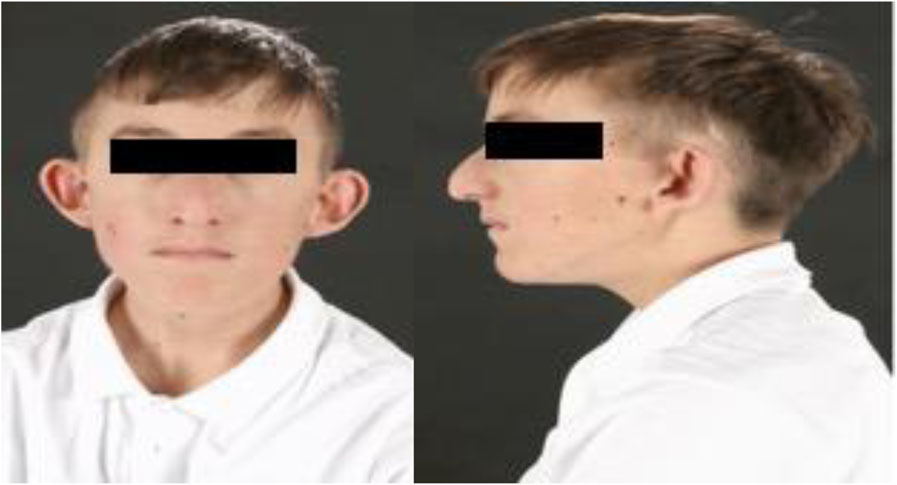
Dysmorphic features: prominent long nasal bridge and forehead, small lower jaw, thin lips, strabismus, down slanting palpebral fissures and low set cupped ears

**Fig. 2:**
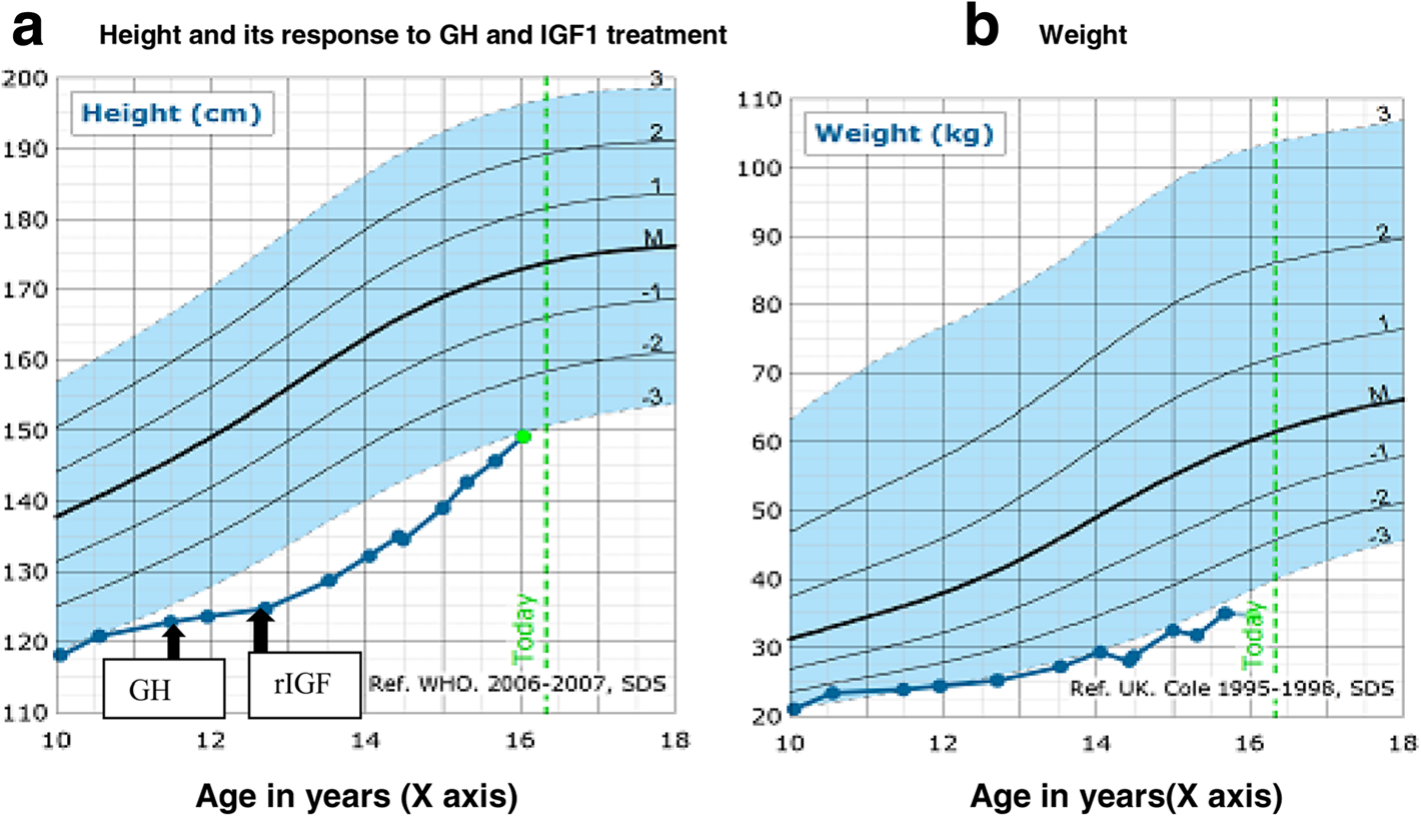
**a** Height and its response to GH and IGF1 treatment. **b** Weight

The patient is a 16-year-old Caucasian British boy born at full term following an induction of labour to non-consanguineous Caucasian healthy British parents. The antenatal scans were normal and the birth weight was 4.1 kg (1.84 SDS). He was admitted to the neonatal unit due to respiratory distress. Whilst in the neonatal unit, he had persistent feeding difficulties and required tube feeding. He was noted to have scaphocephaly that required surgical fixation at 4 months of age. He also developed severe constipation from 5 weeks of age requiring daily bowel washouts from 18 months of age and colostomy at 3 years. He had bilateral undescended testes requiring orchidopexy. He has global developmental delay and complex learning difficulties requiring additional support at school. He also has been diagnosed with autism. At 7 years of age, he was referred to endocrinology for assessment of his severe short stature (−4.11 SDS for height, mid parental height: −1.1 SDS, weight: -2.30 SDS). He has dysmorphic features including prominent long nasal bridge and forehead, small lower jaw, thin lips, low set cupped ears, strabismus and down-slanting palpebral fissures (Fig. 1). He was found to have a normal growth hormone (GH) response (peak GH 11.7 μg/L) (Normal:>6.7 μg/L) to an arginine stimulation test. He had a bone age delay of 3 years and the IGF1 was persistently low at 4.9 nmol/L (−3.2 SDS). TSH (Thyroid stimulating hormone), Free T4 (thyroxine), ACTH (Adreno corticotrophic hormone), prolactin and cortisol concentrations were all within the normal range. A trial of rhGH (recombinant human ﻿growth hormone) (50 μg/kg/day) for a period of 1 year was ineffective in improving height velocity (Fig. 2a). An IGF1 (insulin growth factor-1) generation test after 33 μg/kg of rhGH did not produce any response. Subsequently, recombinant IGF1 (rIGF1) therapy (mecasermin) was commenced at 12.5 years which resulted in improvement of height velocity to -3SDS (Fig. 2a). He has a normal muscle tone and normal deep tendon reflexes. His cranial MRI scan of brain and spine were normal. The hearing has been normal. The echocardiogram and renal ultrasound did not identify any abnormalities. The plasma amino acids, urine organic acids, pyruvic acid analysis were within the normal limits. CGH microarray did not reveal any copy number changes. Targeted sequencing of *IGF1, IGF1R* and *GHR* did not reveal any mutations. Currently, the patient continues to require rIGF1 therapy to support growth. The weight gain continues to be suboptimal (Fig. 2b).

## Material and methods

This study was given favourable ethical opinion by the North West - Liverpool Central Research Ethics Committee (REC Reference: 15/NW/0758) and site study approval was granted by the Clinical Research Business Unit at Alder Hey Children’s NHS Foundation Trust, Liverpool, UK. Informed and written consent was obtained from the parents. DNA was extracted from blood samples of the child and both the biological parents (trio). Exons were captured using SureSelect XT Human All Exon V5 capture library and DNA sequencing was carried out using the Illumina HiSeq4000 at 2 × 150 bp paired-end sequencer. The sequence data were aligned to the reference genome (GRCh37/hg19). The variants present in at least 1% minor allele frequency in 1000 Genomes Project, dbSNP142, and NHLBI ESP exomes were excluded. The predicted deleterious variants included non-synonymous coding, splice site, frameshift, stop gain variants.

## Results

**Fig. 3:**
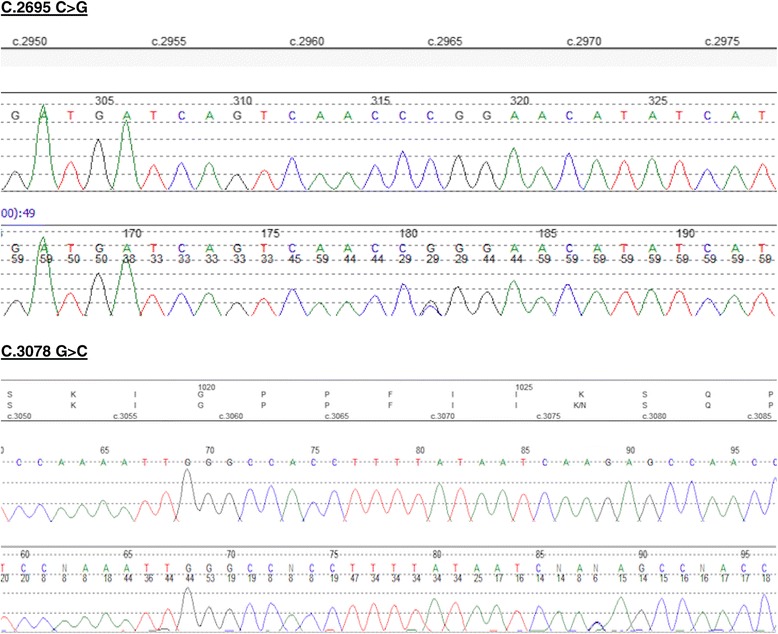
Electropherograms showing the compound heterozygous mutations

**Fig. 4:**
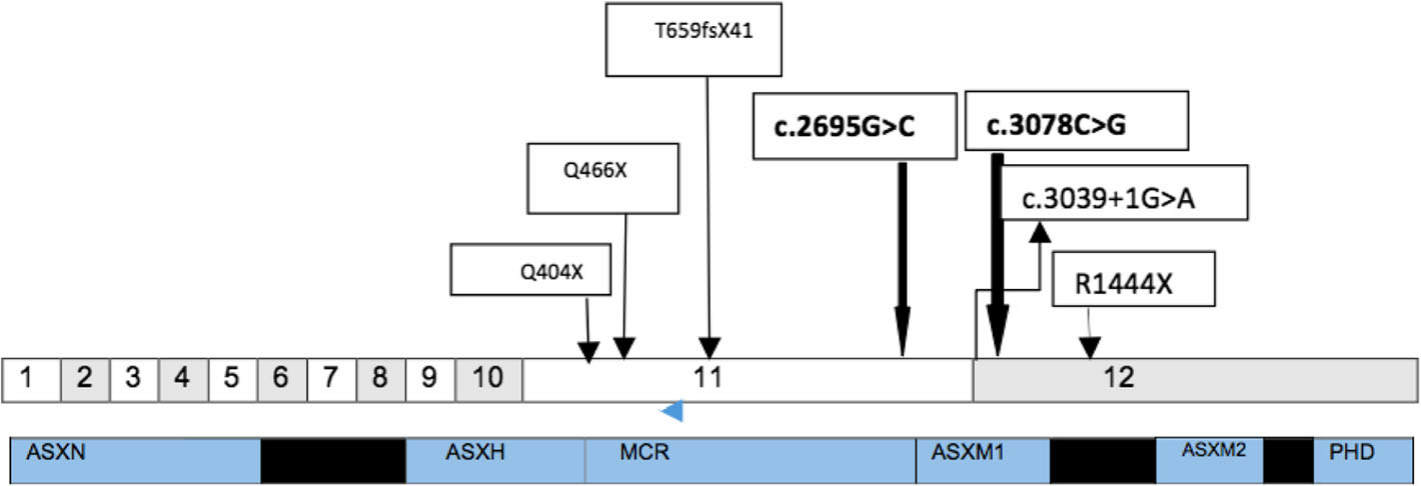
*ASXL3* gene with domains. 1–12 represents the exon numbers. Some of the previously reported mutations (frameshift and truncating) and splice site mutations have been shown. Compound heterozygous mutations in our patient have been highlighted in bold

Two novel heterozygous mutations in *ASXL3* [NM_030632.1]: c.2965C > G, p.R989G inherited from the mother and c.3078G > C, p.K1026 N, inherited from the father were found in the patient. The mutations were subsequently confirmed by Sanger sequencing (Fig. 3). The mutations occur in exon 11 and proximal part of exon 12(Fig. 4). Multiple sequence alignment visualisation using the UCSC Genome Browser showed that both mutated positions are strongly conserved at the protein level across vertebrates as diverse as lemur, bat, fish and frog, implying that mutation could potentially affect the protein structure or function. *In silico* analyses using PolyPhen-2 and SIFT predict the amino acid substitutions to be potentially deleterious to the protein function.

## Discussion

Loss of function mutations in *ASXL3* in the form of de-novo truncating dominant mutations and splicing mutation have been implicated in BRPS. Here we report for the first time, a compound heterozygous *ASXL3* mutation in a patient with BRPS-like features and associated with primary IGF1 deficiency. Pathogenic mutations in *ASXL3* have been reported to occur predominantly in exon 11 and proximal part of exon 12. All the described mutations retain the ASXN and ASXH domains. The compound heterozygous mutations in our patient also lie on exon 11 and proximal exon 12, retaining the ASXN and ASXH domains similar to previously described mutations (Fig. 4). Both these mutations occur on the conserved ASXM1 domain in *ASXL3* (Fig. 4). Both the variants are extremely rare and have a population frequency < 0.01, as indicated from the ExAC browser. The synergistic effect of both of these rare mutations potentially contributes to the loss of function of the protein contributing to the BRPS like phenotype. Our patient has multiple dysmorphic features that overlap with those described in previous reported cases of BRPS such as short stature, failure to thrive, feeding difficulties, cranio-facial features, developmental delay and learning difficulties (Table 1).

**Table 1:** Phenotypic comparison between our patient and other reported patients with BRPS

Phenotype	Our Patient	Bainbridge et al. [1]. 4 patients	Dinwiddie et al. [2]. 1 patient	Srivastava et al. [4]. 3 patients	Hori et al. [3]. 1 patient	Balasubramanian et al. [17]. 12 patients	Kuechler et al. [18]. 6 patients
Clinical							
Feeding problems	+	+	+	+	+	9/12	6/6
Failure to thrive	+	+	+	+	+		3/6
Short stature	+	+		ND	+	2/12	2/6
IUGR	−	3/4	+	2/3	+		−
Craniofacial							
Trigonocephaly	−	1/4	+	1/3	+	ND	ND
Microcephaly	−	2/4	+	−	+	+	1/6
Scaphocephaly	+	−	−	−	−	+	ND
Palate	High arched	1/4	ND	ND	ND	High arched (9/12)	High arched (5/6)
Prominent forehead	+	2/4	ND	1/3	ND	+	5/6
Prominent eyes	−	−	+	−	ND	ND	ND
Palpebral fissures	downslanting		upslanting	downslanting (2/3)	−	downslanting-10/12 Upslanting-2/12	downslanting
Nasal bridge	long	−	depressed	Broad (1/3)	depressed	long, prominent	6/6 (prominent columella)
Low set ears	+	1/4	NA	1/3	−	+	ND
Posteriorly rotated ears	Cupped ears	2/4	+	+	−	+	ND
Anteverted nares	−	+	+	1/3	−	ND	5/6
Small chin	+	ND	ND	2/3	+	+	ND
Ophthalmic							
Strabismus	+	ND	ND	1/3	+	7/12	5/6
Astgmatism	myopia	ND	Myopia (1/3)	Hyperopia (1/3)	myopia	ND	
Neurological							
Developmental delay	+	+	+	+	+	12/12	6/6
Intellectual deficit	+	+	+	2/3	+	12/12	5/6
Seizures	−	−	+	1/3		3/12	2/6
Autism	+	NA	NA	NA	+	9/12	not formally diagnosed
Other Features							
large fontanelle	+	1/4	ND	ND	ND	ND	ND
Undescended testes	+	1/4	ND	ND	ND	ND	ND
Chronic constipation	+	ND	ND	1/3	ND	ND	ND

ND: not described. +: present. -: absent

*ASXL3* belongs to the gene family of ASXL genes, the mammalian homologues of Drosophila Asx. ASXL includes three orthologues: *ASXL1*, *ASXL2* and *ASXL3* that encode the Putative Polycomb group (PcG) protein that has a role in regulating the homeotic genes (Hox) [6]. PcG proteins can act either as transcriptional repressors or activators of Hox genes [6]. The genes in the ASXL family share a common domain architecture consisting of ASXN, ASXH, ASXM1, ASXM2 domains and a PHD finger, and act by forming complexes with other proteins via methylation of histones [4, 6, 7]. *ASXL3* has been implicated in the deubiquitination of histone H2A lysine 119(H2AK119Ub1), a component of the polycomb repressive deubiquitination (PR-DUB) complex [4]. The formation of PR-DUB complex is critical for normal function. *ASXL3* interacts with BAP1, a ubiquitin terminal hydroxylase and removes the mono-ubiquitin (Ub1) from the H2AK119Ub1 [8]. Patients with BRPS have been found to have a significant increase in the H2AK119Ub1 in their fibroblasts because of the impaired deubiquitination [4]. *ASXL3* has a similar expression pattern in human tissues as *ASXL1* but at a relatively lesser levels, which may explain the overlap of some phenotypic features seen in BRPS and BOS [9]. Within the human brain, *ASXL3* expression has been found within the white matter, insula, cingulate gyrus and amygdala [10]. The spinal cord, kidney, bone marrow and liver also express *ASXL3*, but at a lower level when compared to *ASXL1* [9].

Detailed bioinformatics analysis suggests a possible molecular mechanism by which the first of the mutations R989G would lead to a functional defect. A scan against the ELM(Eukaryotic Linear Motif) database shows a stretch of amino-acid residues from the position 989 to 997 within the wild-type *ASXL3* that matches with an interaction motif (LIG_14–3-3_CanoR_1; Accession ELME000417) that describes canonical phosphopeptide binding motif of 14–3-3 group of proteins. 14–3-3 proteins are important cell regulators [11], best known for their role in cell cycle control. The mutated Arginine at position 989 together with a phosphorylated Serine residue, 3–5 residues downstream are the main determinants of interaction with 14–3-3 proteins. These proteins are also characterised as histone modification readers [12]. This links suggestively to the recently determined role of *ASXL3* in histone deubiquitination [4]. According to this hypothesis, mutation of R989 to glycine would prevent the interaction of *ASXL3* with an as-yet unidentified 14–3-3 protein, thereby damaging function through impairing its ability to scaffold epigenetic protein complexes [6]. Although the molecular mechanism of the second mutation K1026 N, is unclear it is possible that this mutation affects phosphorylation of *ASXL3* through its location within recognition motifs for kinases (PIKK group, motif from 1024 to 1030 or GSK3, motif from 1024 to 1031); The molecular defects caused by the two mutations would specify the disorder additively or synergistically by simultaneously impacting on two points of the molecular interaction network of *ASXL3* contributing to its loss of function.

The association of primary IGF1 deficiency in BRPS has not been described before. IGF-1 is a 70-amino acid peptide hormone and growth factor that is structurally homologous to proinsulin [13]. In normal individuals, IGF-1 circulates as part of a ternary complex with a molecular weight of 150 kDa. The complex consists of IGF-1, an acid-labile subunit (ALS), and a protein that binds IGF-1 (IGFBP-3). Primary IGF1 deficiency is defined as basal IGF-1 and height of ≤ -3 SDS with normal or elevated levels of GH [13]. The primary action of IGF1 is mediated by binding to its specific receptor, the insulin-like growth factor 1 receptor (IGF1R), which is present in many tissues. IGF1R is a receptor tyrosine kinase and binding of IGF1 to IGF1R initiates intracellular signalling. IGF-1 is one of the most potent natural activators of the Akt signalling pathway, which stimulates cellular growth and proliferation [14].

Transcriptome analysis of *ASXL3* fibroblasts from patients with BRPS examining the differentially expressed genes (DEGs) has shown that the genes regulating the cellular proliferation are downregulated [4]. IGF1 plays a vital role in activating the Akt signalling pathway, a potent stimulator for cell proliferation and growth [15]. We therefore hypothesise that *ASXL3* potentially has a role in transcriptional activation of *IGF1* involved in this pathway potentially via epigenetic mechanisms [16], which could be contributing to short stature encountered in these patients.

## Conclusions

The compound heterozygous mutations potentially contribute to the loss of function in *ASXL3*, causing a phenotype similar to BRPS. Although with our current knowledge, the molecular interaction between *ASXL3* and *IGF1* is unclear, it may important to look for IGF1 deficiency in the patients with *ASXL3* mutation.

## Consent

Written informed consent was obtained from the patient’s legal guardian(s) for publication of this case report and any accompanying images. A copy of the written consent is available for review by the Editor-in-Chief of this journal.

## Abbreviations

ASXL3: additional sex combs-like 3
BAP1: BRCA1 Associated Protein 1
BOS: Bohring-Opitz syndrome
BRPS: Bainbridge Ropers syndrome
ExAC: Exome Aggregation Consortium
GH: Growth hormone
IGF-1: Insulin Growth Factor-1
SDS: Standard deviation score
Ub1: mono-ubiquitin
